# TRIM29 as a prognostic predictor for multiple human malignant neoplasms: a systematic review and meta-analysis

**DOI:** 10.18632/oncotarget.23617

**Published:** 2017-12-22

**Authors:** Chao Liang, Huiyu Dong, Chenkui Miao, Jundong Zhu, Jie Wang, Pu Li, Jie Li, Zengjun Wang

**Affiliations:** ^1^ State Key Laboratory of Reproductive Medicine and Department of Urology, The First Affiliated Hospital of Nanjing Medical University, Nanjing, China

**Keywords:** TRIM29, prognosis, meta-analysis, malignant neoplasm

## Abstract

Recent studies have shown that tripartite motif-containing protein 29 (TRIM29) had prognostic values in several cancers. However, different studies have been inconsistent. We conducted a meta-analysis to elucidate the precise predictive value of TRIM29 in various human malignant disease. Eleven eligible studies with 2046 patients were ultimately enrolled in this meta-analysis. Heterogeneity between studies was assessed using I^2^ statistics. Pooled Hazard ratios (HRs) with 95% confidence intervals (CIs) for patient survival and disease recurrence were calculated to investigate the correlation between TRIM29 expression and cancer prognosis. The results identified an important link between upregulated TRIM29 expression and poor prognosis in patients with multiple human malignant neoplasms in terms of recurrence-free survival (RFS)/disease-free survival (DFS) (HR = 1.66, 95% CI 1.36–2.04) but favorable progression-free survival (PFS)/metastasis-free survival (MFS) (HR = 0.37, 95% CI 0.16–0.85). We found that high TRIM29 expression predicted no significant impact on overall survival (OS) (HR = 1.32, 95% CI 0.90–1.93). Subgroup analyses showed that high TRIM29 expression predicted poor OS in Asians (HR = 2.21, 95% CI 1.78–2.74) but favorable OS in Caucasian (HR = 0.47, 95% CI 0.25–0.89). TRIM29 might play an essential role in carcinogenesis of multiple human malignant neoplasms and could serve as a biomarker for the prediction of patients’ prognosis.

## INTRODUCTION

Tripartite motif (TRIM) family proteins, most of which have E3 ubiquitin ligase activities, are involved in autophagy, immunity and Carcinogenesis [1–4]. It is likely that autophagy can both suppresses carcinogenesis and promotes tumor progression at different time [5]. Accumulating studies have indicated that TRIM proteins could regulate carcinogenesis positively and negatively [1].

TRIM29, which is also known as ataxia-telangiectasia group D complementing protein (ATDC), is a member of the TRIM family proteins [1, 6]. TRIM29 protein can bind p53 and antagonize p53-mediated functions via inhibition of p53 nuclear activities [5, 7]. Furthermore, TRIM29 functions as a scaffold protein to assemble DNA repair proteins into chromatin followed by efficient activation of DNA damage response. TRIM29 can also reduce acetylation of p53 by promoting the degradation of Tip60 [8]. Following DNA damage, TRIM29 is phosphorylated and interacts with ring finger protein 8 (RNF8), promoting DNA repair [9]. Aberrant expression of TRIM29 facilitates malignant cell growth and inhibit drug-induced apoptosis in bladder cancer, possibly through PKC–NF-κB signaling pathways [10]. It has been also reported that TRIM29 acts as a tumor suppressor through inhibiting Twist-related protein 1 (TWIST1) and suppress epithelial mesenchymal transition (EMT) [11]. Furthermore, it is reported that expression of TRIM29 leads to suppression of anchorage-independent growth (AIG) in multiple cancer cell lines, contributing to poor outcome in cancer patients [12]. These findings suggest that TRIM29 is a multifunctional TRIM protein in the cell differentiation and proliferation. In recent years, TRIM29 was investigated in many clinical studies and was found to have potential prognostic value [13].

TRIM29 has been found to be upregulated in various cancer tissues. Many studies have shown significant associations between high TRIM expression and poor cancer prognosis [14–19], but other studies did not find any significant association [20, 21], and still others showed a negative correlation [20, 22, 23]. Therefore, it is necessary to conduct a meta-analysis to summarize the findings globally and clarify the preliminary predictive value of TRIM29 in tumor prognosis.

In this study, we seek to carry out a meta-analysis to evaluate the overall risk of TRIM29 for survival in patients with cancers. We also discuss the possibility to use TRIM29 as a prognostic marker in terms of clinical practice and statistics.

## RESULTS

### Eligible studies

**Figure 1:**
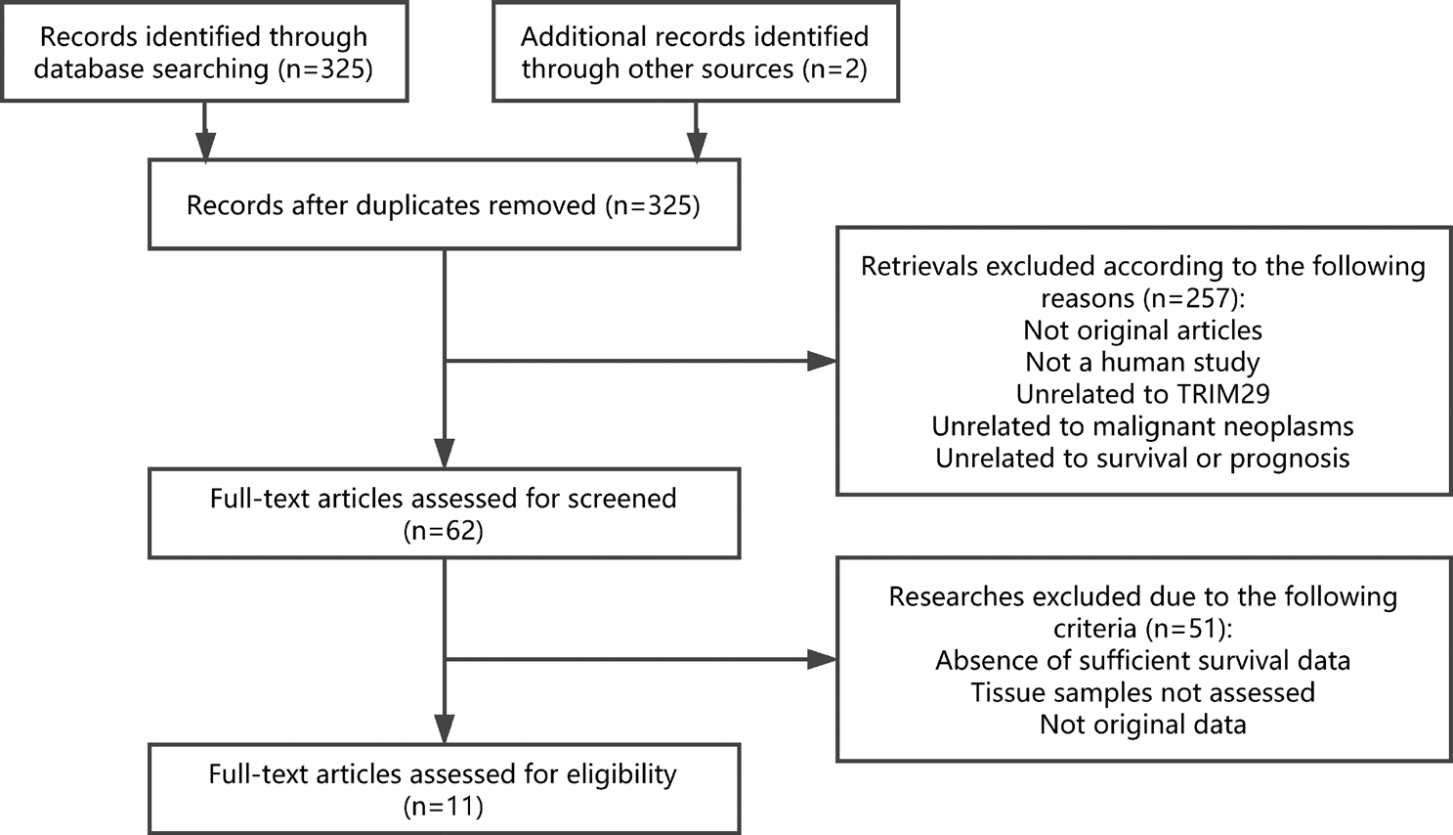
Flow chart of the study selection process Abbreviations: TRIM29, tripartite motif-containing protein 29.

A total of 325 studies were identified from the databases. Based on readings of the article titles and abstracts and according to the inclusion and exclusion criteria, 62 studies were selected for further investigation. Of these 62 candidates, 51 studies were excluded for absence of sufficient survival data, no HRs or survival curve, tissue sample not assessed and not original data. Therefore, 11 articles were ultimately included in the meta-analysis. A flow chart of the study selection process is shown in Figure 1.

### Characteristics of the included studies

The main features of the 13 eligible data are summarized in Table 1 and Table 2. The data collected from the 13 cohorts that included a total of 2046 participants were ultimately included in the meta-analysis. The malignant neoplasms assessed in these studies included cervical cancer, non-small cell lung cancer (NSCLS), oral cavity squamous cell carcinoma, pancreatic cancer, colorectal cancer, bladder cancer, esophageal squamous cell carcinoma, gastric cancer and thyroid carcinoma. Immunohistochemistry (IHC) staining was used to measure TRIM29 expression in 10 studies, while the remaining study used quantitative reverse transcription polymerase chain reaction (qRT-PCR). The pathological types comprised adenocarcinoma, squamous cell carcinoma (SCC) and urothelial carcinoma. These studies were all retrospective in design.

**Table 1:** Main characteristics of studies included in the meta-analysis

First author, publication year	Dominant case nationality	Dominant ethnicity	Malignant disease	Main type of pathology	Detected sample	Survival analysis	Source of HR	Maximum mouths of follow-up
Xu, 2017	China	Asian	Thyroid cancer	Adenocarcinoma	Tissue	DFS/OS	SC	60
Xu, 2016	China	Asian	Cervical cancer	SCC	Tissue	DFS/OS	Reported	141
Song, 2015	China	Asian	NSCLS	SCC/Adenocarcinoma	Tissue	DFS/OS	Reported	72
Harris, 2015	USA	Caucasian	OCSCC	SCC	Tissue	MFS/OS	Reported/SC	120
Harris, 2015	USA	Caucasian	OCSCC	SCC	Tissue	MFS/OS	Reported/SC	120
Sun, 2014	China	Asian	Pancreatic cancer	Adenocarcinoma	Tissue	RFS/OS	Reported/SC	60
Jiang, 2013	China	Asian	Colorectal cancer	Adenocarcinoma	Tissue	DFS/OS	Reported	89
Fristrup, 2013	Denmark	Caucasian	Bladder cancer	Urothelial carcinomas	Tissue	DSS/PFS/OS	Reported/SC	263
Fristrup, 2013	Swedish	Caucasian	Bladder cancer	Urothelial carcinomas	Tissue	PFS/OS	Reported/SC	241
Lai, 2013	China	Asian	Esophageal cancer	SCC	Tissue	OS	Reported	60
Zhou, 2011	China	Asian	NSCLS	SCC	Tissue	OS	Reported	38
Zhao, 2012	China	Asian	Gastric cancer	Adenocarcinoma	Tissue	OS	Reported	78
Kosaka, 2007	Japan	Asian	Gastric cancer	Adenocarcinoma	Tissue	OS	Reported	26.4

OS, overall survival; DFS, disease-free survival; MFS, metastasis-free survival; RFS, recurrence-free survival; PFS, progression-free survival; DSS, disease-specific survival; NSCLC, non-small cell lung cancer; OCSCC, oral cavity squamous cell carcinoma; SCC, squamous cell carcinoma; SC: survival curve.

**Table 2:** HRs and 95% CIs for patient survival or disease progression in association with TRIM29 expression in enrolled studies

First author, publication year	Main assay method	Case number			OS		DFS/RFS		PFS/MFS	
		High expression	low expression	Total	HR (95% CI)	*P* Value	HR (95% CI)	*P* Value	HR (95% CI)	*P* Value
Xu, 2017	qRT-PCR	41	15	56	1.400 (0.680–2.860)	0.026	1.300 (0.540–3.150)	0.030	NM	NM
Xu, 2016	IHC	62	88	150	3.076 (1.544–6.129)	0.001	2.830 (1.495–5.356)	0.001	NM	NM
Song, 2015	IHC	79	241	320	2.102 (1.069–3.193)	0.003	1.384 (0.982–1.952)	0.064	NM	NM
Harris, 2015	qRT-PCR	NM	NM	43	0.210 (1.069–3.193)	0.006	NM	NM	0.024 (0.003–0.909)	0.000
Harris, 2015	IHC	NM	NM	43	0.260 (0.120–0.580)	0.000	NM	NM	0.154 (0.027–0.909)	0.000
Sun, 2014	IHC	109	77	186	2.180 (1.324–4.198)	0.011	1.630 (1.180–2.260)	0.008	NM	NM
Jiang, 2013	IHC	65	86	151	0.431 (0.220-0.841)	0.014	2.370 (1.203–4.651)	< 0.001	NM	NM
Fristrup, 2013	IHC	NM	NM	283	0.810 (0.500–1.290)	0.366	NM	NM	0.600 (0.390–0.930)	0.023
Fristrup, 2013	IHC	NM	NM	576	0.795 (0.505–1.251)	0.321	NM	NM	0.790 (0.510–1.250)	0.321
Lai, 2013	IHC	25	34	59	2.558 (1.435–4.566)	< 0.01	NM	NM	NM	NM
Zhou, 2011	IHC	NM	NM	56	5.300 (1.32–20.774)	0.017	NM	NM	NM	NM
Zhao, 2012	IHC	NM	NM	42	1.950 (1.260–3.030)	0.044	NM	NM	NM	NM
Kosaka, 2007	qRT-PCR	62	62	124	1.050 (0.780–1.440)	0.730	NM	NM	NM	NM

OS, overall survival; DFS, disease-free survival; MFS, metastasis-free survival; RFS, recurrence-free survival; PFS, progression-free survival; DSS, disease-specific survival; NSCLC, non-small cell lung cancer; OCSCC, oral cavity squamous cell carcinoma; SCC, squamous cell carcinoma; HR, hazard ratio; IHC, Immunohistochemistry; qRT-PCR, quantitative reverse transcription polymerase chain reaction; NM: not mentioned; CI, confidence interval; TRIM29, tripartite motif-containing protein 29.

### OS associated with TRIM29 expression

**Figure 2:**
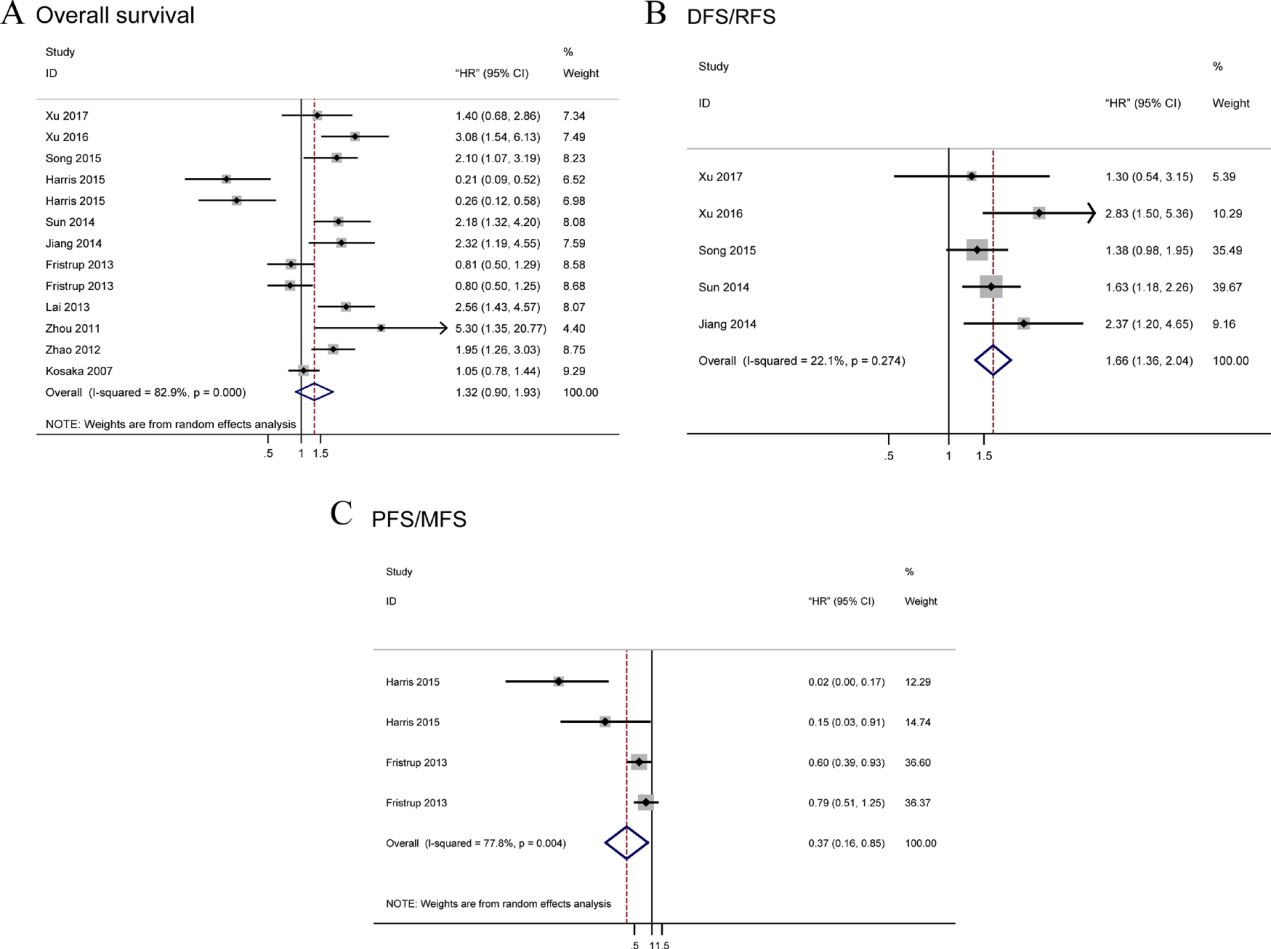
Forest plots of merged analyses for survival associated with TRIM29 expression Notes: (**A**) Forest plot to assess the OS analysis; (**B**) Forest plots for the DFS/RFS analysis; (**C**) Forest plots of PFS/MFS analysis. Abbreviation: OS, overall survival; RFS, recurrence-free survival; DFS, disease-free survival; PFS, progression-free survival; MFS, metastasis-free survival; HR, Hazard ratio.

**Figure 3:**
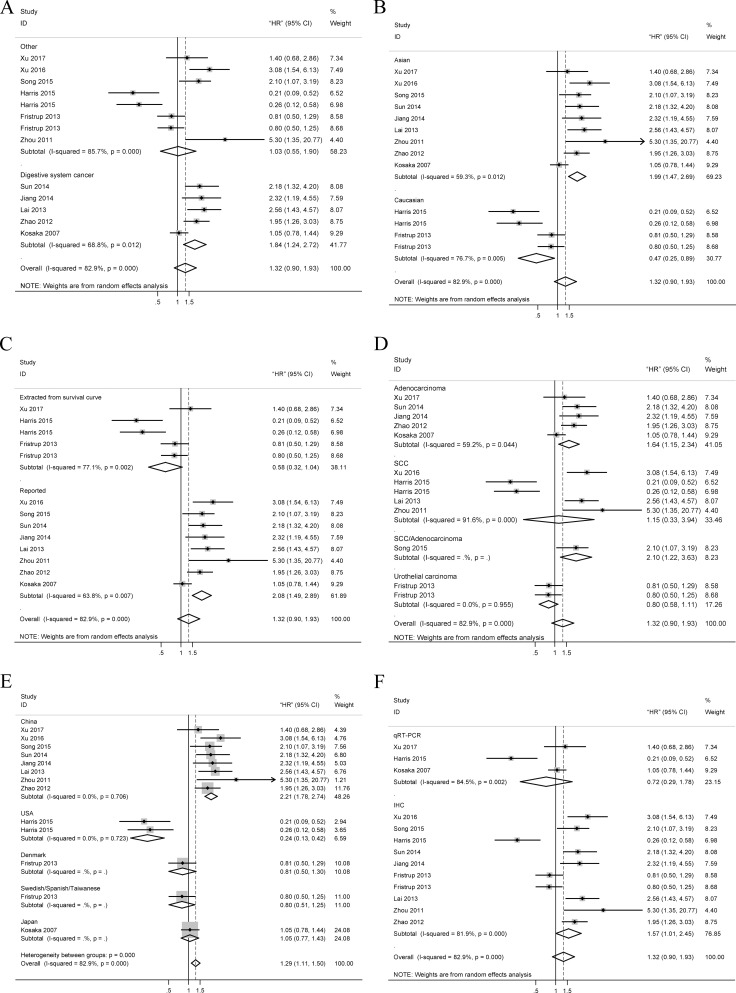
Forest plots of merged analyses for OS associated with TRIM29 expression Notes: (**A**) Forest plots for the subgroup analysis in different tumor types; (**B**) Forest plots for the subgroup analysis in different ethnicities; (**C**) Forest plots for the subgroup analysis in different data sources; (**D**) Forest plots for the subgroup analysis in different pathological types; (**E**) Forest plots for the subgroup analysis in different nationalities; (**F**) Forest plots for the subgroup analysis in different methods. Abbreviations: HR, Hazard ratio; SCC, squamous cell carcinoma; qRT-PCR, quantitative reverse transcription polymerase chain reaction; IHC, immunohistochemistry.

A total of 13 data were used for OS analysis (Figure 2A) with a random-effects model due to significant heterogeneity (*P* = 0.000, *I*^2^ = 84.3%). The result showed that high level of TRIM29 may predict poorer OS, with the pooled HR being 1.32 (95% CI: 0.90–1.93). However, the effect did not reach the level of statistical significance (*P* = 0.161). Stratified analyses were performed by classifying studies into subgroups of case nationality, data source, dominant ethnicity, pathologic type, assay method, tumor site and malignant diseases. In stratified analyses of main malignant type, significant effect was observed between the high level of TRIM29 in digestive system cancer and poorer OS (random-effects model: pooled HR = 1.84; 95% CI: 1.24–2.72; *P* = 0.002) and no significant results were found in other types cancer (random-effects model: pooled HR = 1.03; 95% CI: 0.55–1.90; *P* = 0.000) (Figure 3A). When stratified by the ethnicity, we find a significantly worse OS (random effects model: pooled HR = 1.99; 95% CI: 1.47–2.69; *P* = 0.000) in Asian and a significantly better OS (random effects model pooled HR = 0.47; 95% CI: 0.25–0.89; *P* = 0.021) (Figure 3B) in Caucasian. In subtotal analyses of data source, significant worse OS (random-effects model: pooled HR = 2.08; 95% CI: 1.49–2.89; *P* = 0.000) (Figure 3C) in reported data group was observed and no significant association was found in the other group (random-effects model: pooled HR = 0.58; 95% CI: 0.32–1.04; *P* = 0.070). Subgroup analysis according to pathologic type showed that the combined HR was 1.64 (95% CI: 1.15–2.34; *P* = 0.007) for adenocarcinoma (Figure 3D) and no relationship between SCC and OS (HR = 1.15; 95% CI:0.33–3.94; *P* = 0.827). In subgroup analysis stratified by case nationality, high level of TRIM29 in Chinese people exhibited a significant association with poor OS (HR = 2.21; 95% CI: 1.78–2.74; *P* = 0.000) and no heterogeneity was observed (*I*^2^ = 0.0%, *P* = 0.706) (Figure 3E). USA people conversely exhibited a better OS with high TRIM29 expression (HR = 0.24; 95% CI: 0.13–0.42; *P* = 0.000) (Figure 3E). Finally, the results revealed that high level of TRIM29 significantly associated with worse OS in IHC group (random-effects model: pooled HR = 1.57; 95% CI: 1.01–2.45; *P* = 0.045) and no significant association between OS and qRT-PCR method (Figure 3F).

### Tumor progression and recurrence associated with TRIM29 expression

We analyzed tumor recurrence associated with high TRIM29 expression by combining DFS and RFS. A total of five studies focused on DFS/RFS analysis with low heterogeneity among them (*I*^2^ = 22.1%, *P* = 0.274) (Figure 2B). A fixed-effects model was applied and high level of TRIM29 was significantly related to worse DFS/RFS (pooled HR = 1.66; 95% CI: 1.36–2.04). Additionally, a total of four studies included in the PFS/MFS analysis revealed a protective role of increased TRIM29 expression (pooled HR = 0.37, 95% CI: 0.16–0.85) (Figure 2C), as determined by a random-effects model (*I*^2^ = 77.8% *P* = 0.004).

### Publication bias

**Figure 4:**
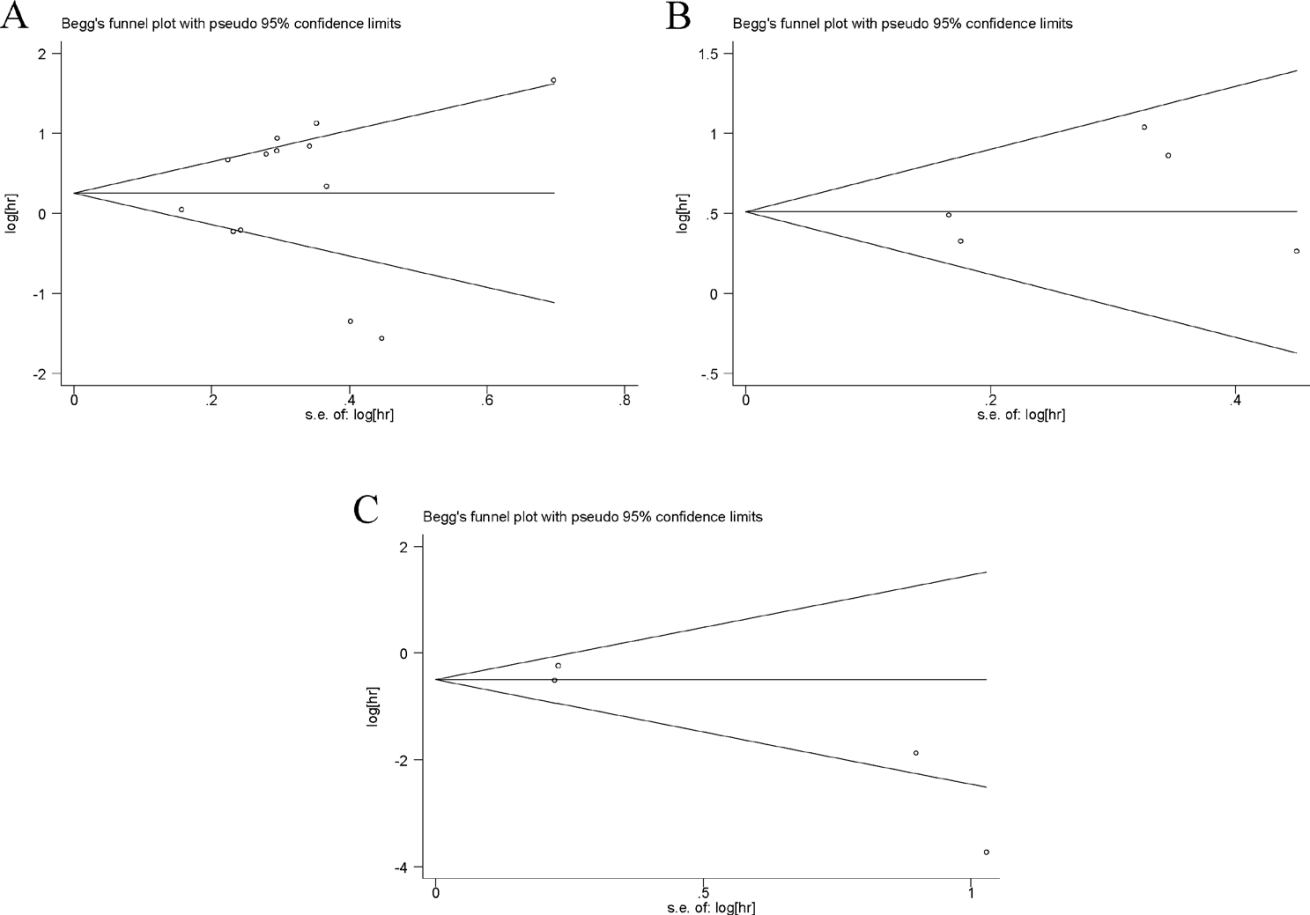
Begg’s funnel plots of the publication bias Notes: (**A**) Begg’s funnel plots of the publication bias for overall merged analysis of OS. Each point represents a separate study. (**B**) Begg’s funnel plots of the publication bias for overall merged analysis of DFS/RFS. (**C**) Begg’s funnel plots of the publication bias for overall merged analysis of PFS/MFS. Abbreviations: OS, overall survival; RFS, recurrence-free survival; DFS, disease-free survival; PFS, progression-free survival; MFS, metastasis-free survival.

Publication bias was detected by Begg’s funnel plot and Egger’s test (Figure 4). Among 13 cohorts evaluating OS, 5 cohorts evaluating DFS/RFS and 4 cohorts evaluating PFS/MFS for TRIM29, no obvious asymmetry was observed in Begg’s funnel plots, and the Egger’s tests also showed no potential publication bias (OS: *P* = 0.778; DFS/RFS: *P* = 0.390; PFS/MFS: *P* = 0.076).

### Sensitivity analysis

**Figure 5:**
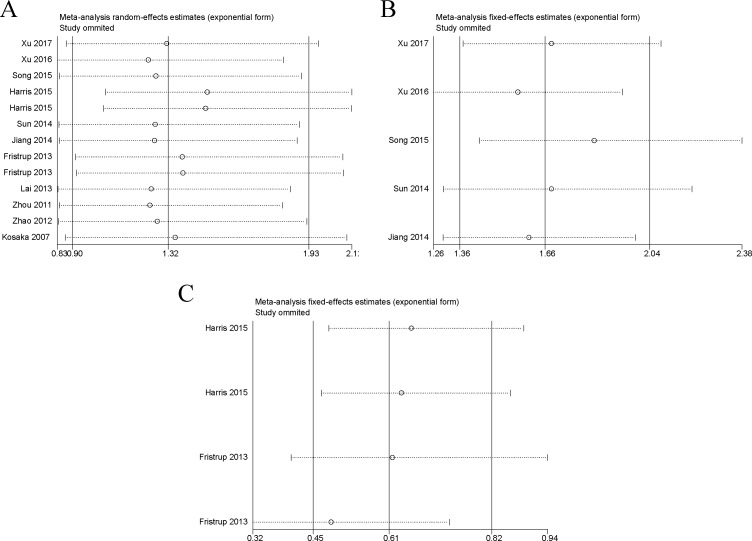
Sensitivity analysis of each included study Notes: (**A**) Sensitivity analysis of OS for individual studies. (**B**) Sensitivity analysis of DFS/RFS for individual studies. (**C**) Sensitivity analysis of PFS/MFS for individual studies. Abbreviations: OS, overall survival; RFS, recurrence-free survival; DFS, disease-free survival; PFS, progression-free survival; MFS, metastasis-free survival.

In the OS, DFS/RFS and PFS/MFS studies, our sensitivity analyses did not indicate alterations in the results due to the inclusion of any individual study (Figure 5), suggesting that no single study significantly influenced the pooled HR or the 95% CI.

## DISCUSSION

TRIM29, also known as ATDC, is a member of the TRIM family of proteins [13]. Increasing evidence showed that aberrant expression of TRIM29 was associated with clinical outcomes for cancer patients. Previous researches have indicated that TRIM29 play important roles in carcinogenesis, autophagy and immunity [13]. TRIM29 also promote tumor cell proliferation via inhibition of p53 nuclear activities [24]. However, it is reported that TRIM29 acts as tumor suppressor in breast cancer through its ability to inhibit TWIST1 and suppress EMT [11]. The role of TRIM29 has been studied extensively in various cancers, but the conclusions are inconsistent.

To our knowledge, the present meta-analysis is the first to systematically analyze the association between TRIM29 expression and clinical features of various cancers, a total of 13 cohorts were included.

Our OS analysis revealed a pooled HR of 1.32, demonstrating that increased TRIM29 expression is associated with a poor outcome; however, this result was not significant (*P* = 0.161). DFS/RFS analysis showed that increased TRIM29 expression is associated with poor outcome significantly (HR = 1.664, *P* = 0.000). On the contrary, the pooled outcome in the PFS/MFS analysis indicated that increased TRIM29 expression is predictive of a better prognosis, with an HR value of 0.365, and this association was also statistically significant (*P* = 0.020). These inconsistent outcomes might hint at dissimilar potential mechanisms that affect cancer recurrence or cancer progression. Recent studies have revealed that TRIM29 exert important effects in tumor cell invasion and metastasis. The TRIM29 have been indicated to regulate EMT by inhibiting expression and activity of the oncogenic transcription factor TWIST1 [11], which may contribute to favorable PFS/MFS when TRIM29 expression is high. However, these results may be insufficiently persuasive because of limited sample size for PFS, MFS, DFS and RFS analysis.

Overall, in subgroup analysis, the results confirmed that relationship between high expression of TRIM29 and prognosis in patients was affected by cancer location, ethnicity of the study subjects, detection method, pathologic type and data source. First, we found that high level of TRIM29 was significantly related to a poor OS in digestive system cancers. This suggested that TRIM29 in these cancers may hint at similar mechanism that interaction between p53 and TRIM29 results in p53 sequestration outside of nucleus. However, while high expression of TRIM29 is associated with worse OS in digestive system cancers, low expression of TRIM29 is associated with favorable OS in multiple tumors, indicating that TRIM29 may also act as a tumor suppressor gene. In the light of this, the prognostic value of TRIM29 may vary in different cancers.

Second, the enrolled studies associated with TRIM29 were grouped into Asians and Caucasians to clarify the impact caused by the different genetic backgrounds on the results. Interestingly, analyses revealed that high TRIM29 expression was a significant worse prediction for OS in Asians, but not in Caucasians. These discrepancies may be attributed to differences in genetic backgrounds and environmental exposures. Third, to find the pathological differences among various cancers, subgroup analysis was performed based on pathological types. It was observed that high level of TRIM29 was significantly associated with worse OS in adenocarcinoma. And we failed to find any statistical significance in other subgroups. These findings suggested that the detection of TRIM29 expression in patients with adenocarcinoma may be useful for prognosis prediction. Finally, to further exclude the impact of method assay, subgroup analysis was performed and a significantly worse OS with high TRIM29 expression was observed in IHC group but not in qRT-PCR group. It revealed that method of IHC was more suitable for predicting patients’ OS.

What calls for special attention is that when interpreting results of meta-analysis, heterogeneity is a potential and crucial issue that cannot be neglected. In this meta-analysis, heterogeneity was observed in comparison for OS and PFS/MFS on TRIM29. This result indicated that the pooled HRs of overall analyses are too crude to present accurate prognostic value of TRIM29. The heterogeneity was largely decreased in some subgroups when we conducted stratified analyses by classifying studies into subgroups of pathological type, sample source, dominant ethnicity, malignant diseases and method assay. Sensitivity analyses were performed and we found that the estimated pooled hazard ratio changed quite a little when successively excluding each single study, which strengthened the results of this meta-analysis. No significant publication bias was shown affecting these possible true results.

It is undeniable that some limitations existed in this meta-analysis. First, the lack of objective standards for evaluating IHC makes it difficult to define a standard cut-off. Most of the current studies have established a score which combine intensity and percentage of IHC as the expression cut-off, and these scores have varied. Therefore, the pooled outcome may be higher or lower than the actual value, which may have caused a bias in the results. Second, the statistical power of the association result of TRIM29 expression and PFS/MFS was reduced because of limited sample size. Third, the number of included studies was not sufficiently large for a comprehensive analysis even though no significant publication bias was detected in the meta-analysis. Fourth, because of the absence of original information, data for survival were extracted from eligible studies based on univariate analysis without adjustment for age, gender, and other risk factors, which may cause confounding bias. Finally, several HRs were calculated based on estimated data extracted from survival curves, some minor differences exist between the exact HRs and the extrapolated data, according to Tierney’s method [25]. These factors should be taken into consideration when drawing a conclusion.

In summary, we concluded that TRIM29 in tumor tissues might be effective indicator for predicting prognosis and tumor progression in the future. Furthermore, TRIM29 was suitable to predict overall survival especially in Asians and digestive system cancers. To get more accurate evaluation of the prognostic role of TRIM29 in patients with cancers, more clinical studies should be carried out before the application of TRIM29 in prognosis of cancer, especially for a single type of cancer.

## MATERIALS AND METHODS

This meta-analysis was strictly performed according to the preferred reporting items of the systematic reviews and meta-analysis (PRISMA) statement and Meta-Analysis of Observational Studies in Epidemiology group (MOOSE) [26, 27].

### Search strategy

Original studies that analyzed the prognostic value of TRIM29 in various cancer were identified by two participants from the PubMed, EMBASE, Web of Science databases. The studies were selected by using the following keywords in various combinations: ‘TRIM29’, ‘ATDC’, ‘ataxia-telangiectasia group D complementing’, ‘tripartite motif containing 29’, ‘carcinoma’, ‘cancer’, ‘death’, ‘incidence’, ‘mortality’ and ‘survival’. The literature published between October 2007 and May 2017 was searched. The relevant references of eligible studies were also searched for additional studies to include.

### Inclusion criteria and exclusion criteria

Studies were considered eligible according to the following criteria: (1) trials was dealing with human cancers, (2) an association between TRIM29 and OS was evaluated, (3) the expression of TRIM29 in tumor tissue was measured (4) HRs could be extracted directly or calculated indirectly in those articles dealing with OS. Articles were excluded if they met the following criteria: (1) hematological malignancies and autoimmune disorders; (2) reviews, case reports, comments, economic analyses, conference abstracts, animal studies, and laboratory studies; (3) lack of crucial information about survival outcome or not being able to estimate HR and 95% CI by the available data.

### Quality assessment

Quality assessment for all the included studies was systematically performed independently by three investigators (Chao Liang, Huiyu Dong and Chenkui Miao). The key points of the current checklist include (1) study population and origin of country; (2) definition of study design; (3) type of carcinoma; (4) clear definition of outcome assessment; (5) definition of measurement of TRIM29 and (6) sufficient follow-up duration. Studies did not satisfy all these six points were excluded so as not to compromise the quality of the meta-analysis.

### Data extraction

All eligible studies were identified by Chao Liang and Huiyu Dong, and uncertain data were reassessed by Chenkui Miao. The data elements of this review including the following: (1) the authors’ names, publication year; (2) The nationality of the studied population; (3) the characteristics of the studied population, including sample type, age, gender, sampling site, tumor type, and pathologic type; (4) detection method, follow-up time; (5) TRIM29 expression levels and cut-off values; and (6) HRs of elevated TRIM29 expression in terms of OS, RFS, disease-specific survival (DSS), MFS, PFS and DFS with 95% CIs and *P* values. If the data were not provided visually and were only provided as Kaplan–Meier curves, the data were extracted from the graphical survival plots, and estimations of the HRs were then performed using a previously described method [25].

### Statistical methods

The HRs and 95% CIs extracted from the eligible articles were combined for survival results. The data were extracted from the graphical survival plots as described above.

Heterogeneity was measured by *Q* statistics as follows: no heterogeneity: 0 < *I*^2^ <25%; low heterogeneity: 25%< *I*^2^ <50%; moderate heterogeneity: 50%< *I*^2^ < 75%; high heterogeneity: 75% < *I*^2^ <100%. If *I*^2^ <50% and *P* > 0.10, a fixed-effect model would be used in combination with HRs, and 95% CI; if *I*^2^ > 50% and *P* < 0.10, then a random-effect model would be selected. Heterogeneity analysis was performed to assess the accuracy of the data, and subgroup and sensitivity analyses were carried out based on professional knowledge. If the subgroup analyses of multiple similar studies still revealed heterogeneity, a random effects model was used [28, 29].

Publication bias was evaluated with a funnel plot and the Egger’s and Begg’s bias indicator test [30]. A two-sided *P* value < 0.05 was considered to indicate statistical significance. All calculations were performed with STATA Statistical Software Version 12.0 (Stata Corp, College Station, TX, USA) and Excel software 2016.
